# Tannic Acid Preferentially Targets Estrogen Receptor-Positive Breast Cancer

**DOI:** 10.1155/2013/369609

**Published:** 2013-11-27

**Authors:** Brian W. Booth, Beau D. Inskeep, Hiral Shah, Jang Pyo Park, Elizabeth J. Hay, Karen J. L. Burg

**Affiliations:** ^1^Institute for Biological Interfaces of Engineering, Clemson University, Clemson, SC 29634, USA; ^2^Department of Bioengineering, Clemson University, Clemson, SC 29634, USA

## Abstract

Research efforts investigating the potential of natural compounds in the fight against cancer are growing. Tannic acid (TA) belongs to the class of hydrolysable tannins and is found in numerous plants and foods. TA is a potent collagen cross-linking agent; the purpose of this study was to generate TA-cross-linked beads and assess the effects on breast cancer cell growth. Collagen beads were stable at body temperature following crosslinking. Exposure to collagen beads with higher levels of TA inhibited proliferation and induced apoptosis in normal and cancer cells. TA-induced apoptosis involved activation of caspase 3/7 and caspase 9 but not caspase 8. Breast cancer cells expressing the estrogen receptor were more susceptible to the effects of TA. Taken together the results suggest that TA has the potential to become an anti-ER^+^ breast cancer treatment or preventative agent.

## 1. Introduction

Breast cancer is the leading cause of cancer deaths among women in developed countries [1, 2]. The use of natural dietary compounds to block or delay the onset of cancer is a promising chemoprevention strategy [3–7]. The purpose of these chemopreventive agents is to suppress cancer cell proliferation, induce cancer cell differentiation, or initiate apoptosis in cancer cells. Studies have demonstrated that natural phytochemicals containing phenolic compounds possess anticancer properties [8–10]. Polyphenols have anticancer functions both *in vitro* and *in vivo* [11–16]. Tannic acid (TA) belongs to the class of hydrolysable tannins and is comprised of a pentagalloylglucose core esterified at all functional hydroxyl groups with gallic acid molecules [17]. TA is a potential anticancer agent. Apoptotic activity is increased in breast cancer and prostate cancer cells in response to exposure to tannin extracts [18–20].

TA functions as a collagen crosslinking agent through both hydrogen bonding and hydrophobic effects. Type I collagen is a common tissue engineering scaffold due to its intrinsically bioactive and biodegradable qualities. Collagen is a naturally derived material and, when uncross-linked, is enzymatically degraded [21]. If used as a biomaterial for tissue engineering purposes the TA-crosslinked collagen Type I would not only serve as an attachment scaffold for cells but also function as an extended release anticancer treatment. As TA-cross-linked collagen is remodeled TA will be released [22].

TA-crosslinked collagen sheets enhance wound healing of the skin in rats [23] and we have previously demonstrated that TA-crosslinked collagen sheets promote adipocyte survival while inducing apoptosis in estrogen receptor-positive (ER^+^) breast cancer cells [24]. Since TA cross-links collagen and has antitumor properties, the combination could prove to be an effective agent to induce localized apoptosis when introduced in tumor environments. If used for tissue reconstruction, TA-crosslinked collagen could provide increased protection against localized tumor recurrence in reconstructed breasts following mastectomy or lumpectomy. The focus of this work is twofold: (1) TA-crosslinked collagen Type I can assume the form of small beads that will, in the long-term, form the basis of an injectable tissue reconstruction scaffold; (2) it investigates the effects of TA on normal human breast epithelial cells and different types of human breast cancer cells lines *in vitro*.

## 2. Materials and Methods

### 2.1. Tannic Acid Cross-Linked Collagen Bead Preparation

A 1 mg/mL collagen type I solution was prepared from a stock solution of 3.1 mg/mL (Advanced BioMatrix, Poway, CA) as described elsewhere [25]. The TA cross-linked beads were prepared using a Nisco Encapsulation Unit VAR V1 electrostatic syringe pump loaded with a 60 : 40 ratio of 1 mg/mL collagen : 1.2% alginate solution in MilliQ water (Sigma-Aldrich, St. Louis, MO); the syringe pump was programmed to pump at 10 mL/h into a 1.5% wt/v CaCl_2_ solution (Fisher, Pittsburgh, PA) in water. One hour after formation, the beads were filtered out using a mesh strainer and placed in TA crosslinking solution comprising TA (10%, 1.0%, or 0.1% wt/v TA/MilliQ water), 1.5% CaCl_2_, 0.15 M NaCl (Sigma-Aldrich), and 1.1% wt/v N-cyclohexyl-2-aminoethanesulfonic acid (Sigma-Aldrich) buffer overnight [26]. Twelve hours later the TA-crosslinked beads were placed in 50 mM sodium citrate (Fisher) for 3 h, washed with deionized (di)H_2_O, and stored in phosphate buffered saline (PBS) at 4°C.

### 2.2. Denaturation Studies

A sample of each concentration of the TA-crosslinked beads was immersed in commercially available blue food coloring for 12 h at room temperature. The dye was used to enhance visualization of the denaturation. Dyed beads were placed individually in diH_2_O and the temperature was gradually increased until the bead morphology was distorted.

### 2.3. Cell Culture

The normal human breast epithelial cell line MCF10A and cell lines isolated from human breast cancers, MCF7 and MDA-MB-231 (all from ATCC; Manassas, VA), were grown in Dulbecco's Modified Eagle Medium (DMEM) with Mammary Epithelial Cell Growth Medium (MEGM) supplements (epidermal growth factor, insulin, bovine pituitary extract, and gentamicin; Lonza; Walkersville, MD) with 10% fetal bovine serum (Atlanta Biologicals; Atlanta, GA) and 1% antibiotic/antimycotic (Life Technologies, Grand Island, NY). The cultures were maintained at 37°C with 5% CO_2_.

For studies using 0.4 *μ*m Transwell inserts (Corning; Corning, NY), the cells were seeded into the individual wells at 30,000 cells/well of 12-well cell culture plates and grown for 24 h. Collagen beads crosslinked with tannic acid at concentrations of 10%, 1.0%, or 0.1% were placed on the Transwell inserts. Each concentration of TA crosslinked collagen beads was placed in three Transwell inserts per plate (400 *μ*L/Transwell insert). One 12-well plate was used for each timepoint (24, 48 h) examined. The lower chamber contained 1 mL of MEGM supplemented DMEM.

### 2.4. Histological Analysis

Cultures of treated and untreated breast cells were washed once with PBS, fixed for 10 min with neutral buffered formalin, washed twice with PBS, and stained with hematoxylin and eosin. All fixation and staining were performed in wells of culture plates.

### 2.5. TUNEL Assay

Apoptotic cells were visualized using Click-iT TUNEL Alexa Fluor Imaging kits from Life Technologies (Grand Island, NY) according to manufacturer instructions. Images were acquired using a Zeiss camera (Thornwood, NY) and the cells were manually counted. All steps were performed in wells of culture plates. Three wells per treatment of each cell line were analyzed. At least 1500 cells per cell line and treatment (i.e., TA concentration) were counted in random fields in each culture well.

### 2.6. Caspase Assays

The SR FLICA Caspase 9 kit and FAM Caspase 3/7 kit from Immunochemistry Technologies, LLC (Bloomington, MI), and the Guava Caspase 8 FAM kit from Millipore (Hayward, CA) were used. Each kit was used according to manufacturers' included instructions.

### 2.7. Western Analysis

Cultures of MCF10A, MCF7, and MDA-MB-231 cells were treated as outlined above. Protein lysates were collected using M-PER Mammalian Protein Extraction Reagent (Thermo Scientific; Rockford, IL) with Halt Phosphatase Inhibitor Cocktail (Thermo Scientific) added. Protein concentration was determined using a Pierce BCA Protein Assay Kit (Thermo Scientific). Lysates were combined with Laemmli's SDS Sample Buffer (Boston BioProducts; Boston, MA) and boiled for 5 min. Proteins were separated using a 4–15% Criterion TGX gel (Bio-Rad), transferred to nitrocellulose, and blocked for 1 h with 1% casein in PBS (Bio-Rad). Membranes were probed with anti-caspase 7 and anti-*β*-actin (1 : 1000; Cell Signaling Technology; Danvers, MA) overnight at 4°C. Membranes were washed thrice with 0.1% Tween-20 in PBS and incubated with HRP-conjugated secondary antibodies (1 : 2500; Cell Signaling Technology) for 2 h at RT. Membranes were washed again with 0.1% Tween-20 in PBS and rinsed in diH_2_O. Chemiluminescence was provided by LumiGLO Reagent (Cell Signaling Technology) and detected using a FluorChem M (Cell Biosciences; Santa Clara, CA).

### 2.8. Statistical Analyses

Each experiment was performed a minimum of three times, with at least three replicates performed within each experiment. Student's *t*-test was used to determine significant differences in denaturation studies. One-way ANOVA was used to analyze cell counts.

## 3. Results

### 3.1. Physical Properties of TA Beads

**Figure 1:**
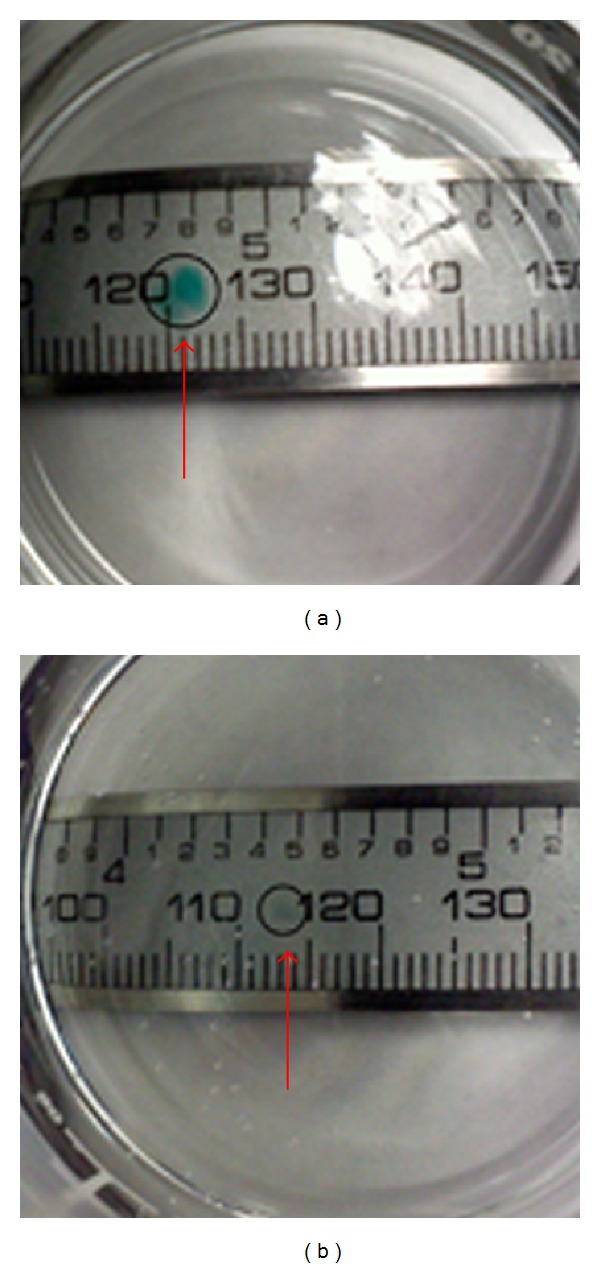
Results of TA bead denaturation studies. TA beads were dyed blue and then subjected to denaturation studies. Beads are in a clear dish over a ruler. (a) Dyed TA bead prior to denaturation; (b) remains of TA bead following denaturation. Arrows indicate bead prior to (a) denaturation or (b) after denaturation.

Samples of the three concentrations of TA-cross-linking collagen type I beads underwent denaturation. The collagen beads were dyed blue for easier visual identification. Once dyed, individual beads were placed in 25 mL of diH_2_O and subjected to increasing temperatures until noticeable shrinkage occurred (Figure 1). The average temperature required for denaturation of the 0.1% TA beads was 58.4°C ± 1.5°C, while for 1.0% TA beads the denaturation temperature was 61.2°C ± 1.3°C and for 10% TA beads the denaturation temperature was 63.8°C ± 1.8°C (Table 1). The denaturation temperature of the 0.1% TA beads was significantly lower than that of both the 1.0% and 10% TA beads (*P* < 0.05). These results are in agreement with previous reports demonstrating that uncross-linked collagen scaffolds have a denaturation temperature of 55°C, while collagen scaffolds crosslinked with 1 mg/mL TA elevated the denaturation temperature to 68°C [24]. The TA-crosslinked collagen beads are stable at human body temperature and therefore have the potential to serve as a growth scaffold for cells during tissue reconstruction.

### 3.2. Effects of Tannic Acid on Normal and Breast Cancer Cells

**Figure 2:**
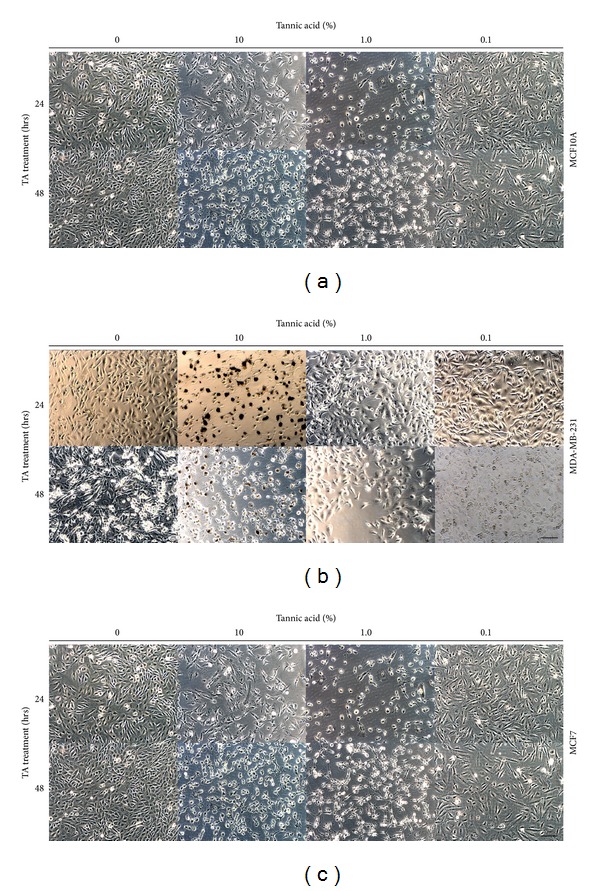
Treatment of human breast cells with tannic acid. Light microscopic images of three human breast cell lines (a) MCF10A, (b) MDA-MB-231, and (c) MCF7; cells were grown to near confluence prior to treatment with 10%, 1.0%, and 0.1% TA cross-linked beads for 24–48 h. Scale bars = 50 *μ*m.

**Figure 3:**
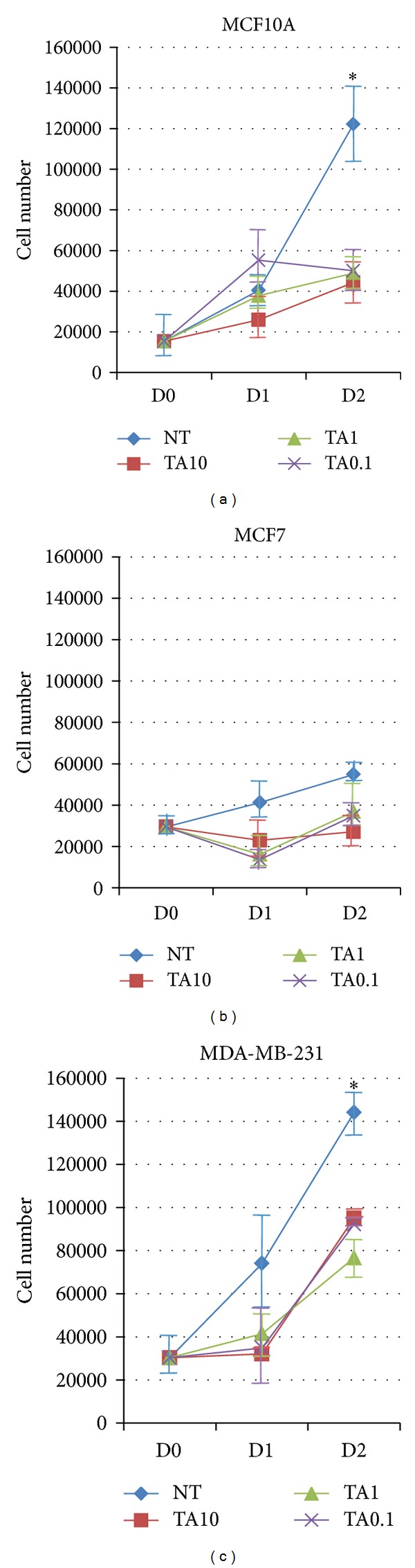
Tannic acid treatment affects cell numbers. Three human breast cell lines (a) MCF10A, (b) MDA-MB-231, and (c) MCF7 were treated with 10%, 1.0%, and 0.1% TA cross-linked beads for 24 and 48 h. The remaining cells were counted. *n* = 3, *P < 0.02, and error bars = SE. NT: no treatment.

The normal human breast epithelial cell line MCF10A and the human breast cancer cell lines MCF7 and MDA-MB-231 were grown in conventional 2D cultures. The breast cell lines were chosen based on their phenotypes. MCF10A cells are immortalized normal breast epithelial cells; MCF7 cells are ER^+^, while the MDA-MB-231 cells are triple negative breast cancer cells (ER negative, progesterone receptor (PR) negative, and HER2 negative) [27–29]. Collagen type I beads crosslinked by various concentrations (10, 1.0, and 0.1%) of TA were added to Transwell inserts placed above the growing cell lines.  Figure 2 illustrates the different effects of the various concentrations of TA on the three cell lines. In cultures of MCF10A cells treated with TA, a change in morphology of the cells treated with the two higher doses of TA (10% and 1.0%) was observed after 24 h, while it was not until 48 h of treatment that a noticeable change in phenotype was observed in the cells treated with the lowest concentration of TA (Figure 2(a)). The changes in observed morphology include rounder cells with fewer protrusions, an increase in the number of detached cells, and an increase in the interstitial space between cells in the cultures. All three concentrations of TA inhibited the growth of the MCF10A cells after 48 h of exposure (Figure 3(a)). The highest concentration of TA used to cross-link the collagen beads had a dramatic effect on the morphology of MDA-MB-231 cells within 24 h of exposure (Figure 2(b)). The lower two concentrations of TA had noticeable effects on the MDA-MB-231 cells after 48 h of treatment. The changes in cellular morphology of the MDA-MB-231 cells were similar to those observed in MCF10A cells, with the addition of brown precipitate seen in the cells exposed to the high concentration of TA. The proliferation of the MDA-MB-231 cultures was inhibited for 24 h after initial exposure to the TA; the cell numbers increased after 48 h but not to that of untreated levels (Figure 3(b)). The effects of TA on MCF7 cells were similar to those seen on MCF10A cells. There was a change in cell morphology, from spindle shaped to more round cells (Figure 2(c)). At the highest concentration of TA, brown precipitate was again observed, as seen in the MCF7 cells.

### 3.3. Tannic Acid Induces Apoptosis in Breast Cells

**Figure 4:**
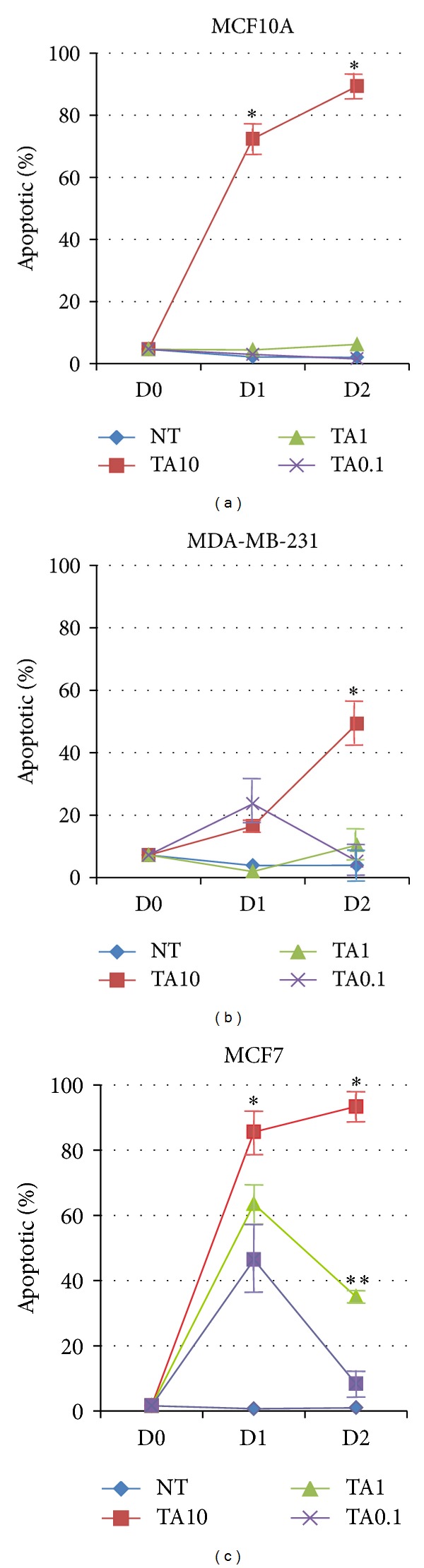
Tannic acid exposure induces apoptosis in breast cells. (a) MCF10A, (b) MDA-MB-231, and (c) MCF7 cells were untreated or treated with 10%, 1.0%, and 0.1% TA cross-linked beads for 24–48 h. TUNEL assays were performed and the results displayed as the percentage of apoptotic cells. *n* = 3, *P < 0.05 versus **P  < 0.05 and error bars = SE. NT: no treatment.

In order to determine if TA reduces cell numbers and alters cell morphology of breast cells by inducing apoptosis, TA-treated cultures of breast cell lines were subjected to TUNEL assays. Previous studies have demonstrated that MCF7 cells are sensitive to the effects of TA [24, 30]. Only the highest concentration of TA, 10%, significantly induced apoptosis in the normal MCF10A cells after 24 h of exposure (Figure 4(a)). Two concentrations of TA induced significant levels of apoptosis in MDA-MB-231 cells compared to untreated MDA-MB-231 cells (Figure 4(b)). MCF7 cells were more sensitive to TA exposure, as all three concentrations induced significant apoptosis within 24 h of exposure and the percentage of apoptotic cells remained significantly higher after 48 h in response to the two higher concentrations of TA, unlike in the other two cell lines (Figure 4(c)). The addition of the highest dose of TA initiated apoptosis in all three cell lines within 24 h of treatment, with the breast cancer cells having a greater number of apoptotic cells when exposed to the lower levels of TA compared to the normal MCF10A cells.

### 3.4. Tannic Acid Induces Apoptosis via Caspase Activation

**Figure 5:**
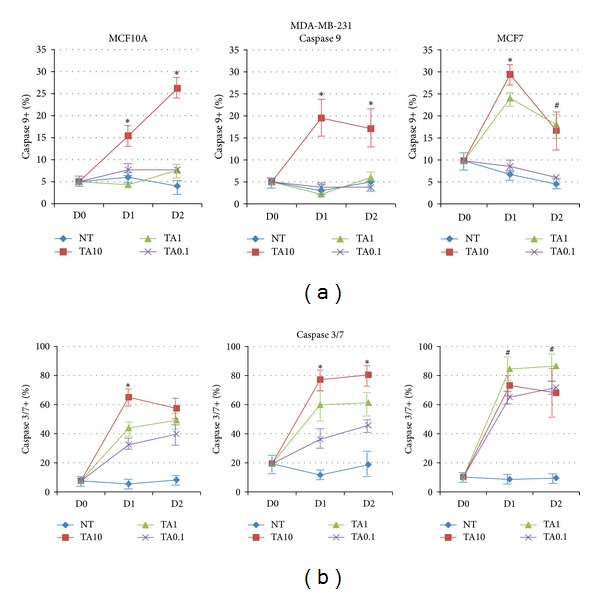
Tannic acid exposure induces apoptosis in breast cells via induction of caspases. MCF10A, MDA-MB-231, and MCF7 cells were untreated or treated with 10%, 1.0%, and 0.1% TA cross-linked beads for 24 and 48 h. Levels of activated (a) caspase 9 and (b) caspase 3/7 were determined using flow cytometry. NT: no treatment. *P < 0.05 TA 10 versus other treatments, ^#^ *P* < 0.05 MCF7 versus MCF10A.

One mechanism involved in the initiation of apoptosis is activation of caspase signaling cascades. Levels of caspase activity were measured using flow cytometry-based caspase activity assays. The levels of activated caspase 9 found in MCF7 cells were significantly higher than the levels observed in MCF10A and MDA-MB-231 cells, for the two highest TA concentrations used after 24 h of exposure (Figure 5(a)). Levels of activated caspase 9 in MCF10A and MDA-MB-231 cells were only significantly elevated in response to the highest concentration of TA. All TA-induced elevated levels of caspase 9 remained elevated after 48 h of exposure.

Levels of the effector caspases 3 and 7 were also investigated. Levels of activated caspase 3/7 were elevated in response to TA exposure in all three cell lines. MCF7 cells are most sensitive to the proapoptotic effects of TA, as significant elevated levels of caspase 3/7 were found in response to all concentrations of TA (Figure 5(b)). The levels of activated caspase 3/7 were also elevated in MDA-MB-231 cells and in MCF10A but not to the extent observed in MCF7 cells. The triple negative MDA-MB-231 cells had significantly higher levels of activated caspase 3/7 in response to the highest dose of TA compared to the MCF10A cells. The flow cytometry-based assay does not discriminate between caspases 3 and 7. Caspases 3 and 7 share 57% sequence homology and have similar substrate preference [31, 32].

**Figure 6:**
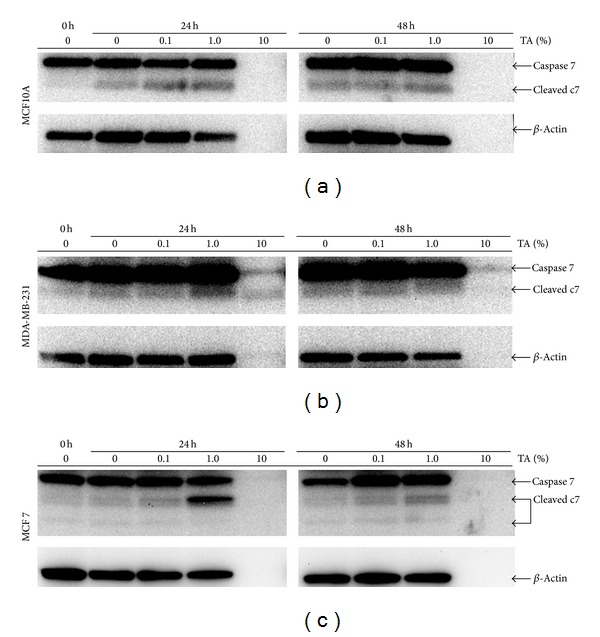
Analysis of caspase 7 in TA-treated breast cells. (a) MCF10A, (b) MDA-MB-231, and (c) MCF7 cells were exposed to TA-crosslinked beads of varying concentrations for the time indicated. Western analysis of collected cell lysates indicated that caspase 7 is activated, as evidenced by the presence of cleaved caspase 7. Increased cleaved caspase 7 is observed in lysates collected from MCF7 cells. *β*-Actin was used as a loading control.

The results of the flow cytometry-based caspase activity assays were confirmed using Western blotting. Since MCF7 cells do not express caspase 3 [33] we focused on caspase 7. We found levels of cleaved caspase 7 elevated in a concentration-dependent manner in MCF10A cells after 24 h of TA exposure (Figure 6(a)). The levels of cleaved caspase 7 remained elevated after 48 h of TA treatment. A similar pattern of elevated cleaved caspase 7 was also observed in cell lysates from MDA-MB-231 cells (Figure 6(b)). Additional forms of cleaved caspase 7 were found in MCF7 cell lysates following TA exposure. Caspase 7 has multiple cleaved forms [34]. The smaller cleaved caspase 7 isoforms were only found in ER^+^ MCF7 cell lysates, not in lysates from the normal MCF10A cells or the triple negative MDA-MB-231 cells. In accordance with the flow cytometry results, the MCF7 cells demonstrated the highest levels of caspase 7 activity in response to TA exposure. According to the protein determination assay, equal amounts of protein were loaded in all lanes. However the *β*-actin loading control demonstrates that this did occur in the lanes labeled TA 10, the highest concentration of TA used. We theorize that the high levels of TA crosslinked additional proteins thereby interfered with the protein concentration determination and protein separation. This did not interfere with flow cytometry as intact cells were analyzed in that assay.

The ER^+^ MCF7 cells were more sensitive to the effects of TA, based on activated caspases 9 and 3/7, as lower concentrations of TA were able to induce elevated levels of the caspases compared to the normal MCF10A cells and the triple negative MDA-MB-231 breast cancer cells.

In all conditions analyzed, the number of cells expressing caspase 8 was less than 1.5%, equal to untreated cells, indicating that caspase 8 is not playing a major role in TA-induced apoptosis (data not shown).

## 4. Conclusions

TA-crosslinked collagen Type I beads are stable at human body temperature and reduce ER^+^ breast cancer cell numbers at a higher rate than normal breast epithelial cells and triple negative breast cancer cells. The reduction in cell number is via induction of apoptosis through activation of caspases 3/7 and 9.

## 5. Discussion

In this study the effects of TA on normal breast epithelial and breast cancer cells were investigated. Previous studies have investigated the effects of TA on one type of breast cancer cell and colon cancer cells [30]. Two breast cancer cell lines, MCF7 and MDA-MB-231, were used in order to determine if the TA affected different forms of breast cancer differently. The two breast cancer cell lines were compared to the MCF10A cell line, representing normal breast epithelial cells. The MCF7 cell line is ER^+^, while the MDA-MB-231 cell line is triple negative (ER^−^PR^−^HER2^−^). Our results indicate that the ER^+^ MCF7 cells were more susceptible to the apoptotic-inducing effects of the TA.

Type I collagen is used in a variety of tissue engineering methods, including breast reconstruction following trauma or mastectomy, due to its intrinsic bioactivity, its biodegradability, and its availability [21]. Uncross-linked collagen Type I is unstable over a period of months, but when a crosslinking agent is added the altered mechanics result in decreased enzymatic and thermal degradability. A leading factor in breast-cancer-related death is the recurrence of a tumor following mastectomy [35, 36]. If an anticancer treatment could be incorporated into a tissue-engineered product, the recurrence of cancer might be reduced. The TA-crosslinked beads used in this study have the potential to be such a tool for breast tissue engineering. The TA-crosslinked beads are stable at body temperature (Table 1) and the released TA possesses anticancer properties. TA-crosslinked collagen sheets are biocompatible and biodurable and have demonstrated a capacity to decrease wound healing time [23]. Taken together, these properties make TA-crosslinked collagen Type I, either as sheets or small injectable beads, a biomaterial with great potential in the fight against breast cancer recurrence.

Multiple epidemiological studies have indicated that diets rich in vegetables and fruits possess anticancer properties. Chemicals present in fruits and vegetables trigger apoptosis in cancer cells and have the potential to be effective in cancer prevention and treatment [3–12]. In most cases, the mechanism of apoptosis induction by these natural substances is unknown.

The caspase (cysteinyl-directed aspartate-specific proteases) family of cysteine proteases are central regulators of apoptosis [37, 38]. TA is known to induce apoptosis via activation of PPAR*γ* [18–20]. PPAR*γ* is cleaved by both caspase 3 and caspase 7 [34]. Apoptosis can be initiated by numerous methods, including activation of cell surface receptors, such as TNF*α* receptor and the Fas receptor, or by extracellular stress that induces DNA damage or mitochondrial damage [39, 40]. Each of these pro-apoptotic pathways uses different members of the caspase family. Caspases 8 and 9 are initiator caspases, while caspases 3 and 7 are involved in cellular breakdown. In this study, TA induced activation of caspase 3/7 and caspase 9, but not caspase 8. Caspase 8 is usually regulated by cell surface receptors such as TNFR and the Fas receptor/CD95; as such, this indicates that TA is not activating a cell surface death receptor. Activation of caspases 3, 7, and 9 can occur due to extracellular stress. Caspase 9 is an initiator caspase that, once activated, cleaves and activates downstream caspases such as 3 and 7. The results show that TA exposure facilitated increased caspase 3/7 and caspase 9 but did not increase caspase 8.

One possible explanation for the increased sensitivity of the ER^+^ MCF7 cells to the effects of the TA may be due to the lack of expression of caspase 3 by MCF7 cells [33]. Both caspase 3 and caspase 7 are downstream of caspase 9 [41] and caspase 3 can act as a feedback regulator of caspase 9 [42]. The lack of caspase 3 within the MCF7 cells may allow unchecked activation of the executioner caspase 7 by the initiator caspase 9. We found levels of both activated caspase 9 and caspase 7 elevated in response to TA.

In conclusion, our results demonstrate a previously undocumented mechanism where TA induces apoptosis in breast epithelial cells via activation of caspases 9 and 7. We demonstrated that the ER^+^ cell line MCF7 was more susceptible to the effects of TA exposure compared to normal MCF10A cells and triple negative breast cancer cells MDA-MB-231. TA crosslinks collagen and collagen is a biomaterial used in breast reconstruction surgeries following mastectomies; thus TA-crosslinked collagen represents a new type of biomaterials that possess anticancer properties. From a clinical perspective this represents a new avenue in the fight against ER^+^ breast cancer recurrence in patients.

## Figures and Tables

**Table 1:** Results of denaturation studies.

% TA	Temp. (°C)
10%	63.8 ± 1.8
1%	61.2 ± 1.3
0.1%	58.4 ± 1.5*

Temperatures are stated as averages of *n* = 5 ± standard deviation.

*P < 0.05.

## References

[1] Parkin DM, Fernández LMG (2006). Use of statistics to assess the global burden of breast cancer. *Breast Journal*.

[2] Desantis C, Siegel R, Bandi P, Jemal A (2011). Breast cancer statistics, 2011. *CA Cancer Journal for Clinicians*.

[3] Manson MM (2003). Cancer prevention—the potential for diet to modulate molecular signalling. *Trends in Molecular Medicine*.

[4] Reddy L, Odhav B, Bhoola KD (2003). Natural products for cancer prevention: a global perspective. *Pharmacology and Therapeutics*.

[5] Fresco P, Borges F, Diniz C, Marques MPM (2006). New insights on the anticancer properties of dietary polyphenols. *Medicinal Research Reviews*.

[6] Shu L, Cheung K-L, Khor TO, Chen C, Kong A-N (2010). Phytochemicals: cancer chemoprevention and suppression of tumor onset and metastasis. *Cancer and Metastasis Reviews*.

[7] Tan AC, Konczak I, Sze DM-Y, Ramzan I (2011). Molecular pathways for cancer chemoprevention by dietary phytochemicals. *Nutrition and Cancer*.

[8] Huang W-Y, Cai Y-Z, Zhang Y (2010). Natural phenolic compounds from medicinal herbs and dietary plants: potential use for cancer prevention. *Nutrition and Cancer*.

[9] Weng C-J, Yen G-C (2012). Chemopreventive effects of dietary phytochemicals against cancer invasion and metastasis: phenolic acids, monophenol, polyphenol, and their derivatives. *Cancer Treatment Reviews*.

[10] Attoub S, Hassan AH, Vanhoecke B (2011). Inhibition of cell survival, invasion, tumor growth and histone deacetylase activity by the dietary flavonoid luteolin in human epithelioid cancer cells. *European Journal of Pharmacology*.

[11] Khan N, Adhami VM, Mukhtar H (2009). Review: green tea polyphenols in chemoprevention of prostate cancer: preclinical and clinical studies. *Nutrition and Cancer*.

[12] Link A, Balaguer F, Goel A (2010). Cancer chemoprevention by dietary polyphenols: promising role for epigenetics. *Biochemical Pharmacology*.

[13] Nguewa PA, Calvo A (2010). Use of transgenic mice as models for prostate cancer chemoprevention. *Current Molecular Medicine*.

[14] Sharif T, Auger C, Alhosin M (2010). Red wine polyphenols cause growth inhibition and apoptosis in acute lymphoblastic leukaemia cells by inducing a redox-sensitive up-regulation of p73 and down-regulation of UHRF1. *European Journal of Cancer*.

[15] Szliszka E, Krol W (2011). The role of dietary polyphenols in tumor necrosis factor-related apoptosis inducing ligand (TRAIL)-induced apoptosis for cancer chemoprevention. *European Journal of Cancer Prevention*.

[16] Yang H, Landis-Piwowar K, Chan TH, Dou QP (2011). Green tea polyphenols as proteasome inhibitors: implication in chemoprevention. *Current Cancer Drug Targets*.

[17] Isenburg JC, Simionescu DT, Vyavahare NR (2005). Tannic acid treatment enhances biostability and reduces calcification of glutaraldehyde fixed aortic wall. *Biomaterials*.

[18] Losso JN, Bansode RR, Trappey A, Bawadi HA, Truax R (2004). In vitro anti-proliferative activities of ellagic acid. *Journal of Nutritional Biochemistry*.

[19] Boitier E, Gautier J-C, Roberts R (2003). Advances in understanding the regulation of apoptosis and mitosis by peroxisome-proliferator activated receptors in pre-clinical models: relevance for human health and disease. *Comparative Hepatology*.

[20] Bawadi HA, Bansode RR, Trappey A, Truax RE, Losso JN (2005). Inhibition of Caco-2 colon, MCF-7 and Hs578T breast, and DU 145 prostatic cancer cell proliferation by water-soluble black bean condensed tannins. *Cancer Letters*.

[21] Glowacki J, Mizuno S (2008). Collagen scaffolds for tissue engineering. *Biopolymers*.

[22] Heijmen FH, Du Pont JS, Middelkoop E, Kreisn RW, Hoekstra MJ (1997). Cross-linking of dermal sheep collagen with tannic acid. *Biomaterials*.

[23] Natarajan V, Krithica N, Madhan B, Sehgal PK (2013). Preparation and properties of tannic acid cross-linked collagen scaffold and its application in wound healing. *Journal of Biomedical Materials Research Part B*.

[24] Cass CAP, Burg KJL (2012). Tannic acid cross-linked collagen scaffolds and their anti-cancer potential in a tissue engineered breast implant. *Journal of Biomaterials Science*.

[25] Vernon RB, Gooden MD, Lara SL, Wight TN (2005). Microgrooved fibrillar collagen membranes as scaffolds for cell support and alignment. *Biomaterials*.

[26] Tebb TA, Tsai S-W, Glattauer V, White JF, Ramshaw JAM, Werkmeister JA (2007). Development of porous collagen beads for chondrocyte culture. *Cytotechnology*.

[27] Cailleau R, Young R, Olive M, Reeves WJ (1974). Breast tumor cell lines from pleural effusions. *Journal of the National Cancer Institute*.

[28] Soule HD, Vazquez J, Long A (1973). A human cell line from a pleural effusion derived from a breast carcinoma. *Journal of the National Cancer Institute*.

[29] Soule HD, Maloney TM, Wolman SR (1990). Isolation and characterization of a spontaneously immortalized human breast epithelial cell line, MCF-10. *Cancer Research*.

[30] Cosan DT, Soyocak A, Basaran A, Degirmenci I, Gunes HV, Sahin FM (2011). Effects of various agents on DNA fragmentation and telomerase enzyme activities in adenocarcinoma cell lines. *Molecular Biology Reports*.

[31] Thornberry NA, Rano TA, Peterson EP (1997). A combinatorial approach defines specificities of members of the caspase family and granzyme B: functional relationships established for key mediators of apoptosis. *Journal of Biological Chemistry*.

[32] Stennicke HR, Renatus M, Meldal M, Salvesen GS (2000). Internally quenched fluorescent peptide substrates disclose the subsite preferences of human caspases 1, 3, 6, 7 and 8. *Biochemical Journal*.

[33] Liang Y, Yan C, Schor NF (2001). Apoptosis in the absence of caspase 3. *Oncogene*.

[34] Boucher D, Blais V, Denault J-B (2012). Caspase-7 uses an exosite to promote poly(ADP ribose) polymerase 1 proteolysis. *Proceedings of the National Academy of Sciences of the United States of America*.

[35] Brewster AM, Hortobagyi GN, Broglio KR (2008). Residual risk of breast cancer recurrence 5 years after adjuvant therapy. *Journal of the National Cancer Institute*.

[36] Maughan KL, Lutterbie MA, Ham PS (2010). Treatment of breast cancer. *American Family Physician*.

[37] Logue SE, Martin SJ (2008). Caspase activation cascades in apoptosis. *Biochemical Society Transactions*.

[38] Taylor RC, Cullen SP, Martin SJ (2008). Apoptosis: controlled demolition at the cellular level. *Nature Reviews Molecular Cell Biology*.

[39] Van Herreweghe F, Festjens N, Declercq W, Vandenabeele P (2010). Tumor necrosis factor-mediated cell death: to break or to burst, that’s the question. *Cellular and Molecular Life Sciences*.

[40] Zakeri Z, Lockshin RA (2008). Cell death: history and future. *Advances in Experimental Medicine and Biology*.

[41] Denault J-B, Eckelman BP, Shin H, Pop C, Salvesen GS (2007). Caspase 3 attenuates XIAP (X-linked inhibitor of apoptosis protein)-mediated inhibition of caspase 9. *Biochemical Journal*.

[42] Srinivasula SM, Ahmad M, Fernandes-Alnemri T, Alnemri ES (1998). Autoactivation of procaspase-9 by Apaf-1-mediated oligomerization. *Molecular Cell*.

